# Histone Deacetylase-2 Expression in Colorectal Cancer: An Immunohistochemical Study and Its Clinicopathological Significance

**DOI:** 10.3390/cancers18091466

**Published:** 2026-05-02

**Authors:** Nikolaos Garmpis, Afroditi Nonni, Dimitrios Dimitroulis, Eleni I. Effraimidou, Anna Garmpi, Miltiadis-Panagiotis Papandroudis, Konstantinos Kontzoglou, Christos Damaskos

**Affiliations:** 1 Department of Colorectal Surgery, Guys and St Thomas’ Hospital, London SE1 7EH, UK; 2 Second Department of Propedeutic Surgery, Laiko General Hospital, Medical School, National and Kapodistrian University of Athens, 11527 Athens, Greece; 3 N.S. Christeas Laboratory of Experimental Surgery and Surgical Research, Medical School, National and Kapodistrian University of Athens, 11527 Athens, Greece; 4 First Department of Pathology, Medical School, National and Kapodistrian University of Athens, 11527 Athens, Greece; 5 First Surgical Department, University Hospital of Alexandroupolis, Democritus University of Thrace, Dragana, 68100 Alexandroupolis, Greece; 6 8th Department of Internal Medicine, Ygeia General Hospital, 15123 Athens, Greece; 7 Medical School, University of Plovdiv, 4002 Plovdiv, Bulgaria; 8 Department of Emergency Surgery, Laiko General Hospital, 11527 Athens, Greece

**Keywords:** colorectal, cancer, clinicopathological, histone, deacetylase, HDAC

## Abstract

**Background/Objectives**: Colorectal cancer (CRC) remains a leading cause of cancer-related mortality and is characterized by substantial molecular heterogeneity, including epigenetic dysregulation. Histone acetylation, regulated by histone acetyltransferases and histone deacetylases (HDAC), has been implicated in CRC development and progression. The aim of the present study was to evaluate HDAC-2 expression in CRC and investigate its association with clinicopathological parameters and patient outcomes. **Methods**: In this retrospective study, tumor tissue samples from 77 patients with CRC and documented recurrence were examined. HDAC-2 expression was assessed by immunohistochemistry and classified as low or high using a semi-quantitative scoring system. Associations with clinicopathological parameters and survival outcomes (disease-free survival, DFS; overall survival, OS) were analyzed. **Results**: High HDAC-2 expression was associated with younger patient age and earlier disease recurrence, while its association with overall survival was borderline. **Conclusions**: HDAC-2 expression may have clinicopathological relevance in CRC, particularly in relation to recurrence-related outcomes, although larger studies are needed to confirm its prognostic significance.

## Simple Summary

Colorectal cancer (CRC) is one of the leading causes of cancer-related deaths worldwide, and its biological behavior can vary greatly between patients. The development and progression of CRC may be influenced by alterations in gene regulation. Histone deacetylases (HDAC) are central regulators of chromatin organization and gene transcription while their dysregulation has been documented across malignancies. This study aims to assess HDAC-2 expression in CRC and to investigate its association with clinicopathological characteristics and patient outcomes. Our findings support further investigation of HDAC-2 as a biomarker in CRC and provide a rationale for future studies exploring its biological and potential translational relevance.

## 1. Introduction

Histone deacetylases (HDAC) serve as critical epigenetic regulators of chromatin dynamics and gene expression, catalyzing the removal of acetyl groups from lysine residues on both histone and non-histone substrates. By modulating chromatin accessibility and the activity of multiple transcriptional complexes, HDAC influence fundamental cellular processes including proliferation, differentiation, cell-cycle control, apoptosis, DNA damage responses, and immune-related signaling [1,2,3,4]. Dysregulation of HDAC expression and activity has been documented across malignancies and is increasingly recognized as a key component of cancer-associated epigenetic reprogramming, contributing to oncogenic transcriptional states and treatment resistance [3,4,5,6,7,8,9]. These observations have established HDAC as pharmacological targets in oncology [2,3,4,5,9,10,11,12,13,14].

**Figure 1:**
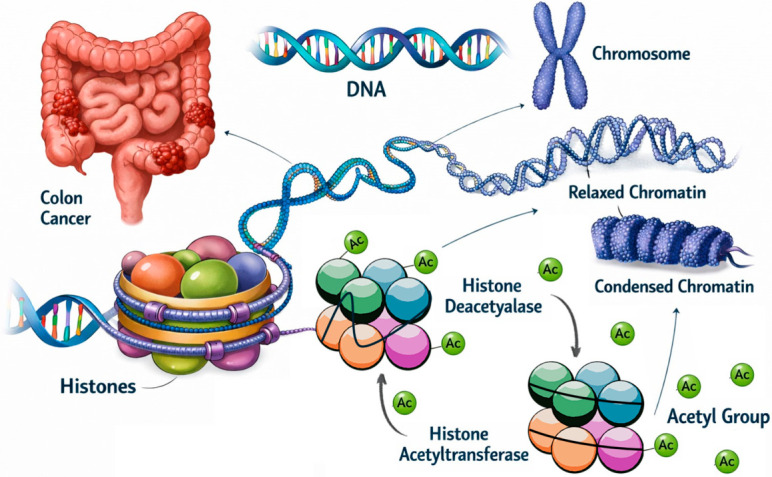
Gene regulation. Histone acetylation and deacetylation are mediated by histone acetyltransferases (HAT) and histone deacetylases (HDAC), respectively.

Histone acetylation represents a dynamic epigenetic mechanism regulated by the opposing activities of histone acetyltransferases (HAT) and HDAC; disruption of this balance has been described in multiple cancer types, including colorectal cancer (CRC) (Figure 1) [2].

HDAC inhibitors (HDACI) have emerged as promising anticancer agents, with established use in selected hematological malignancies and ongoing evaluation in solid tumors [12,13,14,15,16,17,18,19,20,21,22,23,24,25,26,27,28,29,30,31,32,33,34]. Their reported effects include induction of cell-cycle arrest and apoptosis, restoration of tumor suppressor gene expression, and modulation of tumor-associated signaling pathways [3,4,10,35,36,37]. These observations support HDAC modulation as a therapeutically relevant strategy while emphasizing the need for tumor-specific evaluation [10,12,13,14].

A total of 18 human HDAC have been identified, and they are classified into 4 groups according to their structural features and functional roles (Table 1).

Among HDAC family members, class I enzymes (HDAC-2) have attracted particular attention due to their predominant nuclear localization and direct involvement in transcriptional repression complexes [2,4]. HDAC-2 has been proposed as a clinically relevant biomarker and therapeutic target because of its role in regulating gene expression programs associated with malignant phenotypes [38,39,40]. Given the central role of epigenetic deregulation in CRC, evaluation of HDAC-2 in this tumor type appears warranted [7,8].

CRC remains a major public health burden and, in the United States, is reported as the third leading cause of cancer-related death, despite progress in screening and treatment [41]. Although its etiology is not fully clarified, colorectal carcinogenesis is recognized as a multifactorial process shaped by genetic susceptibility, environmental exposures, and a complex molecular background that influences tumor biology and clinical outcome [7,42]. Epigenetic dysregulation in CRC is well documented, including aberrant DNA methylation patterns and related phenotypes such as CpG island methylator phenotype (CIMP), which have been explored for diagnostic and prognostic relevance [6,43,44,45,46]. Histone modifications represent an additional epigenetic layer with potential translational significance in CRC biology and therapy [8]. Early work has suggested that HDAC and HDACI may be particularly relevant to CRC, providing mechanistic and preclinical rationale for targeting this pathway [1,5]. In colorectal neoplasia, increased HDAC-2 expression has been reported across the adenoma-carcinoma sequence, supporting a potential role in early tumorigenesis and progression [47]. Moreover, studies indicate that histone modifiers and their marks may define heterogeneous subsets of colorectal carcinomas and influence responses to HDAC inhibition in vitro, demonstrating the importance of tumor stratification when considering HDAC-targeted approaches [48]. Consistent with this, several reviews have summarized the current evidence and future considerations for HDAC and HDACI in CRC, while emphasizing that the overall clinical evidence base remains encouraging but limited [39,40,49,50].

At the molecular level, CRC most commonly develops through the adenoma–carcinoma sequence driven by chromosomal instability and stepwise accumulation of somatic alterations (e.g., aberrant Wnt signaling following APC pathway disruption, followed by additional changes affecting RAS/MAPK signaling and tumor suppressor pathways). In parallel, a distinct subset of tumors arises through mismatch repair deficiency with microsatellite instability and/or through widespread DNA hypermethylation phenotypes, contributing to biological and clinical heterogeneity. These molecular routes are accompanied by epigenetic reprogramming, including altered DNA methylation and histone modifications, which can influence tumor progression and treatment response [6,7,8,42].

Although HDAC-related pathways have been implicated in CRC biology, the clinicopathological significance of HDAC-2 expression in human CRC tissue remains insufficiently defined, and available immunohistochemical data are limited. Because clinically useful biomarkers ultimately require validation in patient-derived tissue, immunohistochemical assessment of HDAC-2 in CRC may help determine whether its expression is linked to adverse clinicopathological features and recurrence-related outcomes. Therefore, the aim of the present study was to assess HDAC-2 expression in CRC and investigate its association with clinicopathological characteristics and patient outcomes.

## 2. Materials and Methods

### 2.1. Study Population and Clinicopathological Data

The study was carried out at the Medical School of the National and Kapodistrian University of Athens (NKUA), with all participants duly informed and having provided written informed consent prior to enrollment.

**Scheme 1:**
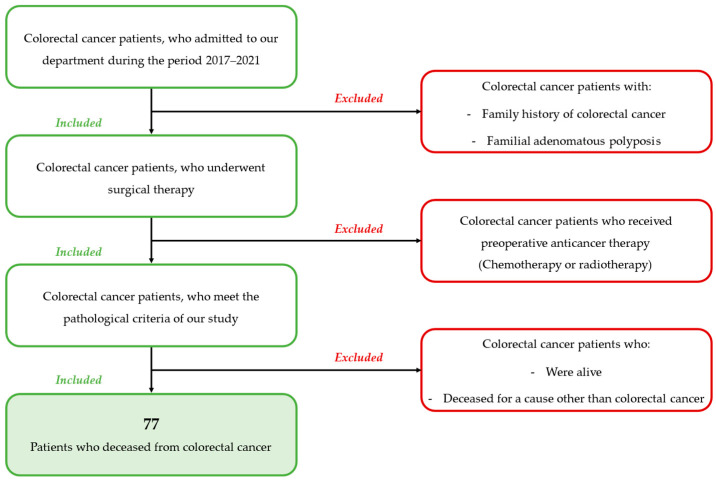
Inclusion and exclusion criteria for patients’ contribution in the study.

In this retrospective study, 77 tissue samples from an equal number of patients with histologically confirmed CRC were included. Cases were identified during the period January 2017–December 2021 according to the predefined eligibility criteria summarized in Scheme 1. Of the included patients, 39 (50.65%) were female and 38 (49.35%) were male. Patients had no known family history of CRC or familial adenomatous polyposis (FAP). The study cohort consisted of consecutive selected CRC cases diagnosed and treated during the period January 2017–December 2021 that fulfilled the predefined eligibility criteria.

The mean age of the patients was 68.3 years (Standard deviation, SD = 12.7). The mean tumor size was 4.6 cm (SD = 2.4). Tumor location was distributed as follows: cecum (20.8%), ascending colon (11.7%), transverse colon (3.9%), descending colon (5.1%), sigmoid colon (37.7%), and rectum (20.8%). All patients underwent surgical treatment, and none received preoperative anticancer therapy (chemotherapy or radiotherapy).

A total of 77 patients with histologically confirmed colorectal cancer who underwent surgical treatment during the study period were included in the analysis. Patients were followed for disease recurrence and survival outcomes. Survival outcomes were assessed using the endpoints disease-free survival (DFS) and overall survival (OS). DFS was defined as the time from surgery to the confirmed disease recurrence, while OS was defined as the time from surgery to death. Normal colonic mucosa specimens without microscopic pathology were used as non-neoplastic reference tissue for immunohistochemical interpretation. These specimens were obtained from individuals who underwent routine screening colonoscopy, in whom biopsy showed no microscopic pathology. The inclusion criteria for patients in the present study are summarized in Scheme 1.

Tumors were staged according to the 8th edition of Tumor, Node, Metastasis (TNM) classification and the American Joint Committee on Cancer (AJCC) staging grouping system [51,52]. TNM-based staging is a useful clinical tool for managing patients with CRC, as accurate stratification is important for determining prognosis and selecting the appropriate therapeutic approach according to disease stage.

Based on these criteria, 9 patients were stage IIA (11.7%), 19 stage IIIA (24.6%), 25 stage IIIB (32.5%), 15 stage IIIC (19.5%), and 9 stage IVA (11.7%). The stage distribution of the cohort does not reflect that of an unselected CRC population and should be interpreted in the context of the retrospective eligibility criteria and case availability.

Aforementioned patient characteristics are summarized in Table 2 and Table 3.

### 2.2. Immunohistochemical Staining and Evaluation

Immunohistochemical staining for HDAC-2 was performed on formalin-fixed, paraffin-embedded CRC tissue specimens. A mouse monoclonal anti-HDAC-2 antibody was used (Histone Deacetylase 2 (HDAC-2) Antibody (C-8): sc-7899, Santa Cruz Biotechnology, Dallas, TX, USA). The antibody recognizes an epitope corresponding to amino acids 435–488 at the C-terminus of HDAC-2.

For assessment of immunohistochemical staining intensity, non-neoplastic colonic mucosa samples were used as reference tissue. These specimens were obtained from individuals who underwent routine screening colonoscopy, in whom biopsy showed no microscopic pathology. The non-neoplastic colonic mucosa samples were formalin-fixed, paraffin-embedded, and processed using the same immunohistochemical staining protocol as the tumor specimens.

Paraffin tissue blocks were sectioned at 4 μm. Sections were deparaffinized in xylene and rehydrated through graded alcohols, followed by rinsing in water. Antigen retrieval was carried out using microwave heating in 10 mM citrate buffer (pH 6.1) for 15 min at maximum power, following the manufacturer’s protocol. Endogenous peroxidase activity was suppressed by incubating the sections in freshly prepared 0.3% hydrogen peroxide in methanol for 30 min at room temperature in the dark. Non-specific binding was blocked using Background Sniper (Biocare Medical, Concord, CA, USA) for 5 min, followed by a 10 min buffer wash.

Tissue sections were incubated with an anti-HDAC-2 antibody for 1 h at room temperature in phosphate-buffered saline (PBS) at a dilution of 1:200. This was followed by incubation with a biotinylated linker (Biocare Medical) for 10 min at room temperature and subsequent application of streptavidin–horseradish peroxidase (Biocare Medical). Immunoreactivity was detected using 3,3′-diaminobenzidine (DAB) (Vector Laboratories, Newark, CA, USA) for 10 min. Sections were counterstained with Harris hematoxylin and mounted using Entellan (Merck, Darmstadt, Germany). Negative controls were prepared by omitting the primary antibody. As an external technical positive control, breast cancer tissue sections with previously validated HDAC-2 expression were used. We acknowledge that a CRC-specific positive control would be preferable for tissue-matched validation [39,40].

Evaluation of HDAC-2 immunostaining was performed independently by three observers blinded to the clinicopathological data, and discrepancies were resolved by consensus. At least 1000 tumor cells were counted per case, and cases were considered HDAC-2-positive when ≥5% of tumor cells showed positive staining. Assessment was performed using light microscopy.

The HDAC-2 expression score was calculated as the product of two semi-quantitative scoring scales, the extent of tumor cell positivity (0–4) and the corresponding staining intensity (0–3). Finally, HDAC-2 expression was categorized as low for scores ranging from 0 to 6 and high for scores between 8 and 12. The aforementioned scales are described in Table 4. The cutoff was predefined according to the semi-quantitative scoring system used in this study, with scores 0–6 representing absent to weak expression and scores 8–12 representing moderate to strong expression.

As a marker of cellular proliferation, Ki-67 immunoreactivity was quantified as the percentage (%) of positively stained tumor nuclei. The mean Ki-67 value was 25.8% (SD 15.8) (Table 3), and cases were categorized as low (below the mean) or high (above the mean).

### 2.3. Statistical Analysis

Quantitative variables were summarized as mean ± SD or median with interquartile range (IQR), as appropriate, while categorical variables were presented as counts (N) and percentages (%). Comparisons of categorical data were conducted using Pearson’s chi-square test or Fisher’s exact test. For continuous variables, between-group differences were analyzed using Student’s *t*-test. Relationships between continuous variables were examined using Spearman’s rank correlation coefficient (r) and one-way ANOVA, as applicable. Survival analysis was performed using the Kaplan–Meier method, with differences between curves assessed by the log-rank test. All statistical tests were two-sided, and a *p*-value < 0.05 was considered statistically significant. Statistical analyses were conducted using GraphPad Prism (v10.0, GraphPad Software, San Diego, CA, USA). No multivariable survival modeling was performed in the present study.

## 3. Results

**Figure 2:**
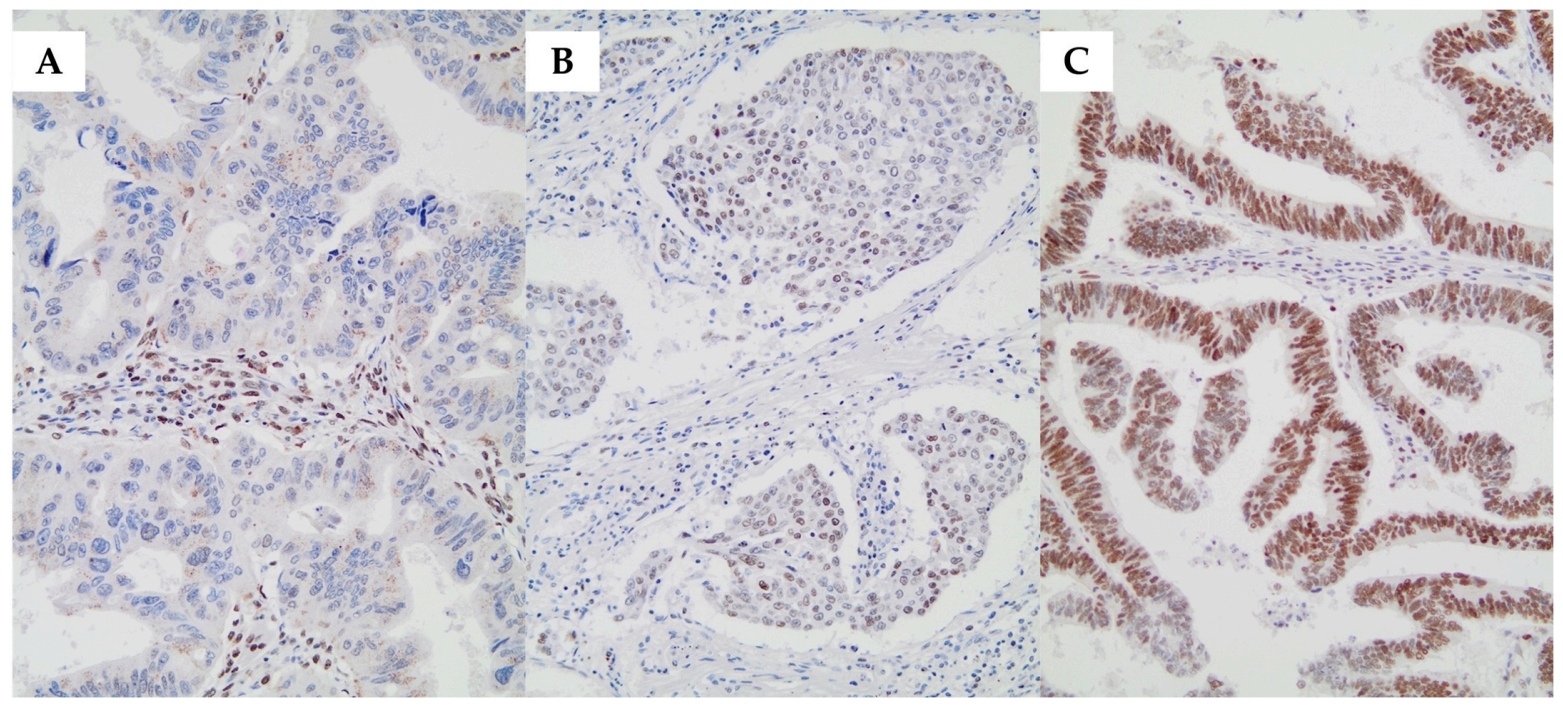
(**A**) Colorectal cancer tissue section showing negative HDAC-2 immunostaining (×200). (**B**) Colorectal cancer tissue section showing low HDAC-2 immunostaining with nuclear localization (×200). (**C**) Colorectal cancer tissue section showing high HDAC-2 immunostaining with nuclear localization (×200).

Among the 77 CRC cases, 4 (5.2%) showed negative HDAC-2 staining, whereas 73 (94.8%) demonstrated positive HDAC-2 immunoreactivity. In all positive cases, HDAC-2 was located in the nucleus. Representative CRC sections with negative, low, and high HDAC-2 immunostaining are shown in Figure 2. In non-neoplastic colonic mucosa, HDAC-2 staining was [absent/minimal/weak and nuclear], whereas CRC specimens more frequently showed stronger nuclear expression. Representative images of normal mucosa have been added.

Based on the semi-quantitative scoring system described above, 28 cases (36.4%) exhibited low HDAC-2 expression, and 49 cases (63.6%) exhibited high HDAC-2 expression. These data are summarized in Table 5.

Associations between HDAC-2 expression category (low vs. high) and clinicopathological/laboratory parameters, including age, sex, tumor size, tumor location, disease stage, and Ki-67 are presented in Table 6 and Table 7.

Patients with high HDAC-2 expression were younger on average than those with low HDAC-2 expression (mean age 66.8 vs. 70.9 years, *p* = 0.0112). Although the association reached statistical significance, the effect size was small and the scatter in the data was considerable.

**Scheme 2:**
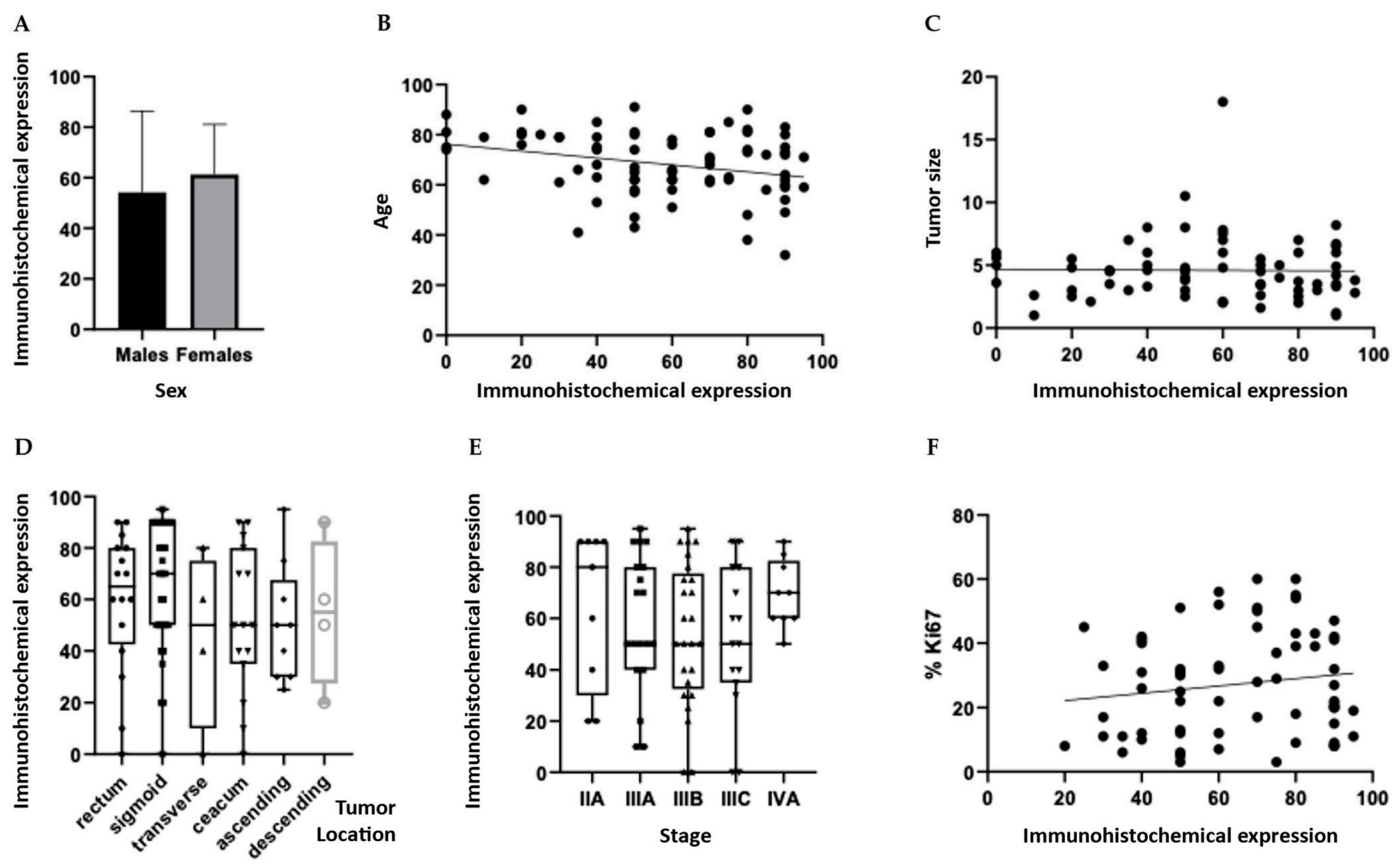
(**A**) HDAC-2 immunohistochemical expression score according to sex. (**B**) Correlation between HDAC-2 immunohistochemical expression score and patient age (R^2^ = 0.08262; *p* = 0.01125). (**C**) Correlation between HDAC-2 immunohistochemical expression score and tumor size. (**D**) HDAC-2 immunohistochemical expression score according to tumor location (cecum, ascending colon, transverse colon, descending colon, sigmoid colon, rectum). (**E**) HDAC-2 immunohistochemical expression score according to disease stage. (**F**) Correlation between HDAC-2 immunohistochemical expression score and Ki-67. In the box-and-whisker plots, the box represents the interquartile range (IQR), the horizontal line indicates the median, and the whiskers represent the minimum and maximum values. Circles denote outliers.

In the following Scheme 2, HDAC-2 immunohistochemical expression is presented in relation to patient sex (Scheme 2A) and age (Scheme 2B), tumor size (Scheme 2C) and location (Scheme 2D), disease stage (Scheme 2E), and the proliferation marker Ki-67 (Scheme 2F).

**Scheme 3:**
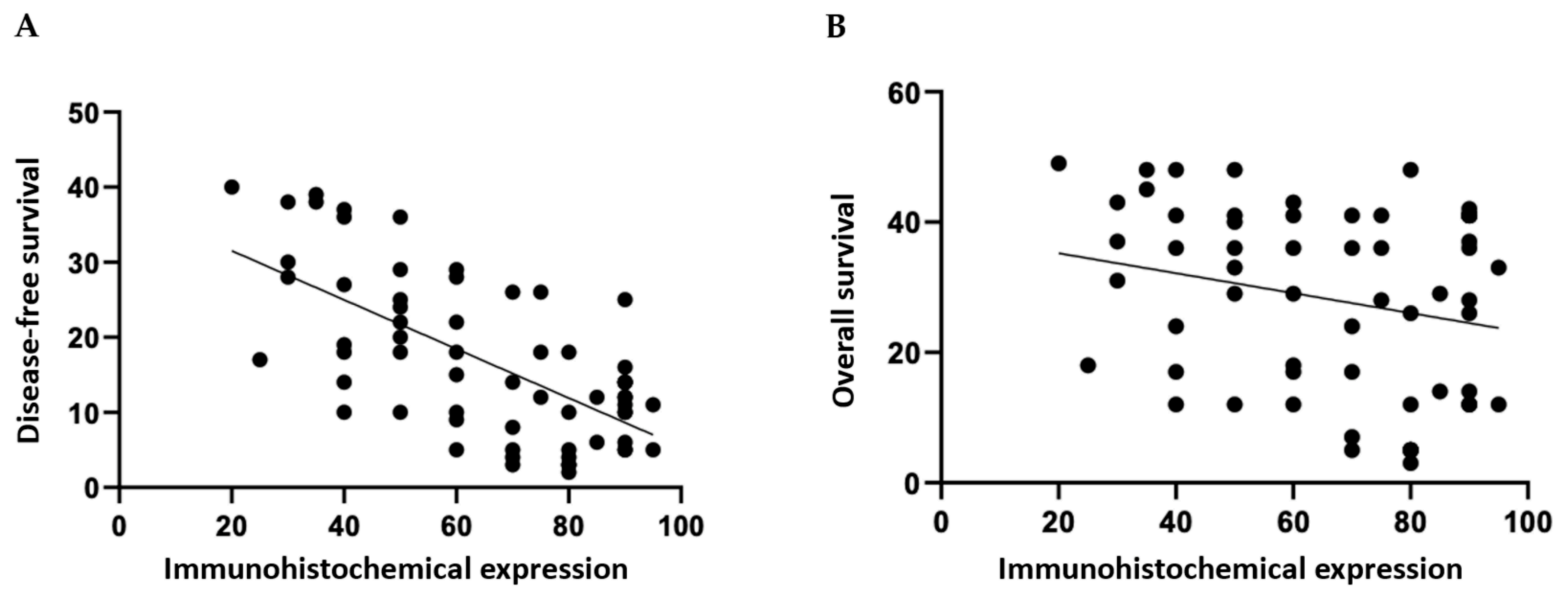
(**A**) Correlation between HDAC-2 immunohistochemical expression score and disease-free survival (DFS). (**B**) Correlation between HDAC-2 immunohistochemical expression score and overall survival (OS).

The mean DFS was 16.6 months (SD = 9.8), with a median of 14 months, whereas the mean OS was 29.9 months (SD = 13.4), with a median of 36 months. Higher HDAC-2 immunohistochemical expression was significantly associated with earlier disease recurrence (log-rank test, *p* < 0.0001) (Scheme 3A) and showed a borderline association with shorter overall survival (log-rank test, *p* = 0.0552) (Scheme 3B).

**Scheme 4:**
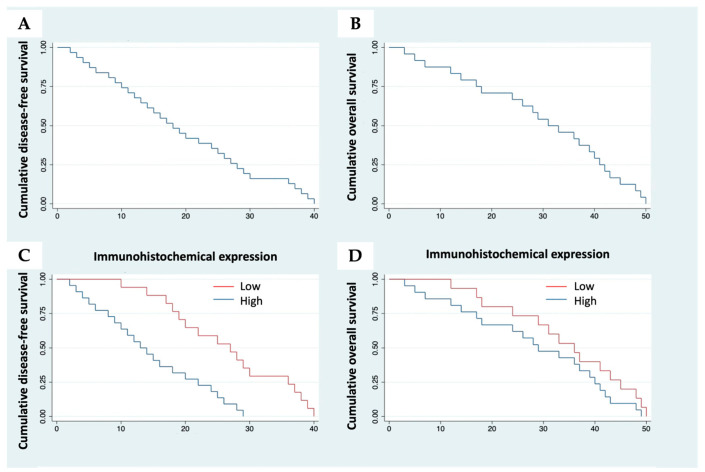
Kaplan–Meier curves (**A**) Disease-free survival in the overall cohort. (**B**) Overall survival in the overall cohort. (**C**) Disease-free survival stratified by HDAC-2 immunohistochemical expression (low vs. high). (**D**) Overall survival stratified by HDAC-2 immunohistochemical expression (low vs. high).

Kaplan–Meier survival curves for DFS and OS overall, are presented in Scheme 4 (Scheme 4A,B). It was also found that a higher degree of HDAC-2 immunohistochemical expression was significantly associated with earlier disease recurrence (log-rank test, *p* < 0.0001). In addition, higher HDAC-2 immunohistochemical expression was borderline associated with shorter overall survival (log-rank test, *p* = 0.0552). The Kaplan–Meier survival curves for DFS and OS, according to the level of HDAC-2 immunohistochemical expression, are shown in the following Diagram (Scheme 4C,D).

## 4. Discussion

In this retrospective immunohistochemical study, HDAC-2 expression was detected in the majority of CRC specimens and was consistently localized to the nucleus. Within the analyzed cohort, high HDAC-2 expression was associated with younger patient age and earlier disease recurrence, whereas the association with overall survival was only borderline. Taken together, these findings suggest a possible relationship between HDAC-2 expression and recurrence-related disease behavior in CRC. At the same time, the strength of this interpretation must remain limited by the selected nature of the cohort, the absence of multivariable modeling, and the lack of mechanistic validation.

The available literature on HDAC-2 expression in CRC remains limited and somewhat heterogeneous. Our findings are broadly consistent with the concept that HDAC-related dysregulation accompanies colorectal tumor progression, as increased HDAC-2 expression has been described across the adenoma-carcinoma sequence [47]. This supports the view that HDAC-2 upregulation may represent a biologically relevant feature of colorectal neoplasia rather than an incidental finding. However, our results differ from those of Ashktorab et al., who did not identify significant clinicopathological correlations for HDAC-2 expression in colorectal neoplasia [47]. Such differences may reflect not only biological heterogeneity but also important methodological variation, including differences in cohort composition, enrichment for aggressive disease, stage distribution, tissue handling, antibody selection, antigen retrieval conditions, scoring cutoffs, and statistical power. In addition, previous work suggests that histone modifiers and their associated marks may define biologically distinct CRC subsets and may influence responses to HDAC inhibition [48]. Taken together, the existing evidence suggests that HDAC-2 expression is unlikely to behave uniformly across all CRC populations and should therefore be interpreted in light of both tumor heterogeneity and methodological context.

The association between high HDAC-2 expression and earlier recurrence observed in the present study is biologically plausible in light of the known functions of HDAC-2 in transcriptional repression and chromatin remodeling. Increased HDAC-2 expression may reflect a broader epigenetic state favoring tumor cell survival, proliferative persistence, adaptive stress responses, and reduced expression of genes involved in growth control, differentiation, and apoptosis. In CRC, where epigenetic deregulation is a major contributor to tumor heterogeneity, HDAC-2 upregulation may therefore mark tumors with a more aggressive and recurrence-prone phenotype. This interpretation is also consistent with the wider literature suggesting that altered histone-modifying activity can accompany malignant progression and may influence biological behavior beyond simple histological classification. Nevertheless, because the present study did not include mechanistic assays, these interpretations remain hypothetical. The findings should therefore be understood as clinicopathological associations that generate biological hypotheses rather than prove causal involvement of HDAC-2 in CRC progression.

The age-related finding also warrants careful interpretation. Patients with high HDAC-2 expression were younger on average than those with low expression, but the effect size was small and the scatter in the data was considerable. Accordingly, although the association reached statistical significance, its explanatory power appears limited. This may indicate a weak biological relationship, a cohort-specific feature, or the influence of unmeasured confounders. It would therefore be inappropriate to place major biological weight on age alone. Instead, this result should be regarded as a secondary observation that requires validation in larger and more representative cohorts.

From a methodological standpoint, the present study has several important limitations that influence interpretation. First, the retrospective design restricts causal inference. Second, the cohort size is modest, which may limit the stability of the observed associations, particularly for overall survival. Third, the analyzed population was not representative of the broader CRC population. Because the cohort was restricted to patients with recurrence and CRC-related death, it was enriched for aggressive disease biology. This introduces substantial selection bias and limits generalizability. As a result, the observed association between high HDAC-2 expression and earlier recurrence should be interpreted within this selected subgroup rather than extrapolated directly to all patients with CRC. In this sense, the present findings are best viewed as preliminary and hypothesis-generating.

A further limitation is the absence of multivariable survival modeling. Although high HDAC-2 expression was associated with earlier recurrence in the present cohort, the current analysis does not establish whether HDAC-2 is an independent prognostic factor or instead reflects confounding by other clinicopathological variables such as disease stage, tumor location, or proliferative activity. This issue is particularly relevant because recurrence dynamics in CRC are influenced by multiple interacting variables. Future studies should therefore incorporate Cox proportional hazards modeling or similar multivariable approaches in order to determine whether HDAC-2 retains prognostic relevance after adjustment for established clinicopathological factors.

Additional caution is required because immunohistochemical assessment is inherently semi-quantitative. Although scoring was performed independently by three observers blinded to clinicopathological data and discrepancies were resolved by consensus, some degree of observer-related variability cannot be excluded. Formal interobserver agreement statistics were not calculated, which represents a methodological limitation. More quantitative approaches, such as digital pathology-based image analysis, could improve reproducibility in future studies. Similarly, external transcriptomic or protein-level validation in independent datasets would provide a useful complementary perspective and would strengthen confidence in the biological relevance of the observed staining patterns.

Another source of uncertainty relates to the broader biological context of HDAC regulation in CRC. Potential modifiers of epigenetic regulation, including diet, metabolic status, environmental exposures, and naturally occurring HDAC-modulating compounds, were not available in this retrospective dataset. Given the established relevance of diet and metabolism to CRC biology, such factors may contribute to interpatient variability in HDAC-2 expression and clinical outcome. Although they could not be evaluated here, they should be considered in future prospective biomarker studies.

The study also does not include functional validation. Therefore, the data do not establish a causal role for HDAC-2 in CRC progression and are insufficient to support HDAC-2 as a therapeutic target on the basis of this analysis alone. Functional studies will be required to determine whether HDAC-2 actively contributes to aggressive tumor behavior or instead reflects broader epigenetic dysregulation associated with aggressive disease. Approaches such as knockdown or overexpression models, proliferation and invasion assays, and validation in independent clinical cohorts or public datasets would substantially strengthen the biological interpretation of the present findings.

Despite these limitations, the present study still contributes clinically oriented evidence suggesting that HDAC-2 expression is associated with recurrence-related outcomes in CRC. This may be relevant in the broader context of evolving cancer treatment strategies [53]. Modern oncology has progressively moved from predominantly surgical and cytotoxic approaches toward more molecularly targeted, immune-based, and biomarker-informed frameworks. In this landscape, epigenetic therapies occupy a distinct position because they seek to modulate dysregulated transcriptional and chromatin states rather than single mutated oncogenic drivers. Histone deacetylase inhibitors already have established activity in selected hematological malignancies and continue to be explored in solid tumors, including CRC, although their precise role remains incompletely defined. Within this broader therapeutic context, biomarkers that help identify biologically meaningful epigenetic states may eventually become relevant to future translational strategies.

From a broader translational perspective, these findings may be viewed within the continuing evolution of cancer treatment from predominantly surgical and cytotoxic approaches toward targeted, immune-based, and biomarker-informed strategies. Recent overviews of the therapeutic landscape have emphasized that modern oncology increasingly depends on identifying biologically meaningful vulnerabilities, including epigenetic dysregulation, that may complement traditional molecular classifications. In this context, epigenetic therapies occupy a distinct position because they aim to modulate aberrant transcriptional and chromatin states rather than single mutated oncogenic drivers. Histone deacetylase inhibitors already have established activity in selected hematological malignancies and continue to be investigated in solid tumors, including CRC, although their exact role remains incompletely defined. Within this broader framework, biomarkers associated with specific epigenetic states may eventually become relevant to translational study design and patient stratification.

However, this point must be stated cautiously. The present study does not provide evidence for treatment selection and does not demonstrate that patients with high HDAC-2 expression are more likely to respond to HDACI. Nor does it establish that HDAC-2 itself is a therapeutically actionable driver in CRC. What the present data do support is a more modest conclusion: that HDAC-2 expression may represent a clinically relevant biomarker candidate associated with recurrence-related disease behavior in a selected CRC cohort and that this observation justifies further investigation in broader and methodologically stronger settings.

Overall, the present findings place HDAC-2 within a biologically and clinically relevant area of CRC epigenetics, but they should be interpreted with appropriate restraint. Validation in larger, unselected, prospectively assembled or independent CRC cohorts, ideally incorporating multivariable analysis and functional confirmation, will be necessary to clarify whether HDAC-2 has true independent prognostic significance and whether its relevance extends beyond association into translational utility.

## 5. Conclusions

To the best of our knowledge, the present study is among the first to demonstrate that increased HDAC-2 expression in CRC is potentially associated with clinically meaningful parameters, including patient age, disease recurrence, and OS. Although high HDAC-2 expression was associated with earlier recurrence and showed a borderline association with overall survival, these findings should be interpreted cautiously given the limited cohort size and potential selection bias. Further studies in larger and more representative CRC populations are required to determine whether HDAC-2 has true independent prognostic value.

In this study, high HDAC-2 expression in CRC was associated with clinicopathological features, particularly earlier disease recurrence, within the analyzed cohort. These findings support the potential relevance of HDAC-2 as a biomarker of disease behavior, but they should be interpreted cautiously given the retrospective design and the characteristics of the study population.

Overall, the present study suggests that HDAC-2 expression may have clinicopathological relevance in CRC, particularly in relation to recurrence-related outcomes within the analyzed cohort. However, because of the retrospective design, selected patient population, and lack of functional or therapeutic-response data, these findings should be interpreted cautiously. Further validation is required before HDAC-2 can be considered a robust biomarker or linked to therapeutic decision-making in CRC.

## Figures and Tables

**Table 1:** Classification of the human histone deacetylase (HDAC) family.

Classification			Member
Classical	Class I		HDAC-1 HDAC-2 HDAC-3 HDAC-8
	Class II	A	HDAC-4 HDAC-5 HDAC-7 HDAC-9
		B	HDAC-6 HDAC-10
Sirtuins	Class III		SIRT-1 SIRT-2 SIRT-3 SIRT-4 SIRT-5 SIRT-6 SIRT-7
Classical	Class IV		HDAC-11

HDAC: Histone deacetylase; SIRT: Sirtuin.

**Table 2:** Quantitative clinicopathological parameters.

Parameter	Mean Value (M)	Standard Deviation (SD)
Age	68.3	12.7
Size (cm)	4.6	2.4
Ki67	25.8	15.8

**Table 3:** Stage distribution of the cohort.

Stage	Mean Value (M)	Standard Deviation (SD)
IIA	9	11.7
IIIA	19	14.6
IIIB	25	32.5
IIIC	15	19.5
IVA	9	11.7

**Table 4:** Scoring system for the evaluation of HDAC-2 immunohistochemical expression.

Proportion of positive stained cells	0	<10	≥10 & <33	≥33 & <66	≥66
	0	1	2	3	4
Staining intensity	Negative	Positive			
		Low	Median		High
	0	1	2		3
Immunohistochemical expression	Low		High		
	0–6		8–12		

**Table 5:** Distribution of HDAC-2 immunohistochemical expression in the study cohort.

Immunohistochemical Expression	Low	High
Number (N)	28	49
Percentage (%)	36.4	63.6

**Table 6:** Association of HDAC-2 immunohistochemical expression with patient age and tumor size.

Immunohistochemical Expression		Low	High	*p*
Age	M	70.9	66.8	0.0112
	SD	11.8	13	
Size (cm)	M	4.9	4.4	0.8955
	SD	3.1	1.8	

M: Mean value; SD: Standard deviation.

**Table 7:** Association of HDAC-2 immunohistochemical expression (low vs. high) with sex, tumor location, disease stage, and Ki-67.

Immunohistochemical Expression		Low		High		*p*
		N	%	N	%	
Sex	Female	12	15.6	27	35.0	0.2478
	Male	16	20.8	22	28.6	
Location	Cecum	4	5.2	12	15.6	0.5446
	Ascending	5	6.5	3	3.9	
	Transverse	1	1.3	2	2.6	
	Descending	3	3.9	1	1.3	
	Sigmoid	7	9.1	22	28.6	
	Rectum	8	10.4	9	11.6	
Stage	IIA	2	2.6	7	9.1	0.8537
	IIIA	8	10.4	11	14.3	
	IIIB	9	11.6	16	20.8	
	IIIC	7	9.1	8	10.4	
	IVA	2	2.6	7	9.1	
Ki67	Low	16	20.8	25	32.4	0.2329
	High	12	15.6	24	31.2	

## Data Availability

The data presented in this study are available on request from the corresponding author due to privacy and ethical restrictions. The data are not publicly available as they contain information related to human participants.

## Abbreviations

The following abbreviations are used in this manuscript:

HDAC	Histone deacetylase
HDACI	Histone deacetylase inhibitor
HAT	Histone acetyltransferase
CRC	Colorectal cancer
CIMP	CpG island methylator phenotype
NKUA	National and Kapodistrian University of Athens
SD	Standard deviation
DFS	Disease-free survival
OS	Overall survival
TNM	Tumor, Node, Metastasis
AJCC	American Joint Committee on Cancer
PBS	Phosphate-buffered saline
DAB	3,3-diaminobenzidine
IQR	Interquartile range
